# Multifaceted Stoichiometry Control of Bacterial Operons Revealed by Deep Proteome Quantification

**DOI:** 10.3389/fgene.2019.00473

**Published:** 2019-05-24

**Authors:** Jing Zhao, Hong Zhang, Bo Qin, Rainer Nikolay, Qing-Yu He, Christian M. T. Spahn, Gong Zhang

**Affiliations:** ^1^Key Laboratory of Functional Protein Research of Guangdong Higher Education Institutes, Institute of Life and Health Engineering, Jinan University, Guangzhou, China; ^2^Institut für Medizinische Physik und Biophysik, Charité – Universitätsmedizin Berlin, Berlin, Germany

**Keywords:** operons, proteome quantification, HRM-MS, multifaceted stoichiometry control, mass-spectrometry, DIA, translation

## Abstract

More than half of the protein-coding genes in bacteria are organized in polycistronic operons composed of two or more genes. It remains under debate whether the operon organization maintains the stoichiometric expression of the genes within an operon. In this study, we performed a label-free data-independent acquisition hyper reaction monitoring mass-spectrometry (HRM-MS) experiment to quantify the *Escherichia coli* proteome in exponential phase and quantified 93.6% of the cytosolic proteins, covering 67.9% and 56.0% of the translating polycistronic operons in BW25113 and MG1655 strains, respectively. We found that the translational regulation contributes largely to the proteome complexity: the shorter operons tend to be more tightly controlled for stoichiometry than longer operons; the operons which mainly code for complexes is more tightly controlled for stoichiometry than the operons which mainly code for metabolic pathways. The gene interval (distance between adjacent genes in one operon) may serve as a regulatory factor for stoichiometry. The catalytic efficiency might be a driving force for differential expression of enzymes encoded in one operon. These results illustrated the multifaceted nature of the operon regulation: the operon unified transcriptional level and gene-specific translational level. This multi-level regulation benefits the host by optimizing the efficiency of the productivity of metabolic pathways and maintenance of different types of protein complexes.

## Introduction

An operon is a cluster of genes transcribed in a single mRNA. This principle is conserved across bacterial and archaeal genomes, as well as mitochondria and chloroplast (Wolf et al., 2001; Price et al., 2005; Zheng et al., 2005). Operons are also found in virus and some lower eukaryotes, including yeasts, nematodes, and insects (Blumenthal, 2004; Ben-Shahar et al., 2007; Osbourn and Field, 2009; Pi et al., 2009; Gordon et al., 2015). In a typical bacterial genome, more than half of the protein-coding genes are organized in multigene operons. A classical bacterial operon generates an mRNA strand with polycistronic structure containing multiple coding sequences and are translated together in the cytoplasm. These genes are often of related functions, for example, to build a protein complex or to participate in one metabolic pathway. Therefore, grouping related genes as operons under the control of a single promotor is often thought to simplify the regulation of gene expression for rapid adaptation to environmental changes.

An intuitive presumption of the operon organization is to maintain stoichiometry of the gene products. It was argued that co-regulation could be evolved by merging two independent genes in proximity together under the control of the same promoter, to reduce the control complexity (Lawrence and Roth, 1996; Osbourn and Field, 2009), Li et al. (2014) measured protein synthesis rates by using ribosome profiling and implied that the synthesis rates quantitatively might reflect the stoichiometry of the protein complexes. Studies showed that an operon with one complex promoter might be better than two independent promoters; organization of genes in operons substantially reduces the shortfall in production of complex-forming individual proteins (Iber, 2006; Osbourn and Field, 2009). However, recent advances of omics techniques raised counter-arguments. A transcriptome-level study revealed that certain adjacent genes within one operon are not similarly transcribed in *M. pneumoniae*. In half of the polycistronic operons, genes exhibited a decaying expression according to its rank in the operon, which is termed “staircase-like decay behavior” (Guell et al., 2009). Considering the widespread post-transcriptional regulations including translational control and protein turnover (Schwanhausser et al., 2011), it is still under intensive debate whether this “staircase-behavior” influences the protein abundance (Maier et al., 2011; Schmidt et al., 2011; Arike et al., 2012).

Theoretically, proteins in a complex should follow the stoichiometry, while the proteins involved in the same pathway may need differential expression controls (Guell et al., 2009). For example, the enzymes in various amino acids synthesis pathways are regulated in single-input modules (SIMs). A series of such enzymes are successively expressed in one operon (Zaslaver et al., 2004; Seshasayee et al., 2006). Meanwhile, the different catalytic kinetics of these enzymes determines that these enzymes should not be expressed at the same quantity (Zaslaver et al., 2004). These genes tend to duplicate to evolve a larger gene regulatory network (Teichmann and Babu, 2004), indicating their regulation is less stringent, and an operon arrangement might be unnecessary. Therefore, a more detailed proteome-wide and quantitative investigation is necessary to discover the scope and impact of the operons in gene expression regulation.

A method capable to assess a quantification of the proteome should be used in this case. Although stable isotope labeling methods are more accurate than label-free mass-spectrometry (MS) methods (Arike et al., 2012), the isotopes may affect the physiology of the bacteria (Xie and Zubarev, 2015). The isotope labeling is more suitable for comparative quantification of multiple samples than estimating abundance of the proteins within one sample (Neilson et al., 2011). Therefore, label-free MS methods should be used. Arike et al. (2012) compared three label-free quantification methods (iBAQ, emPAI, and APEX) and found a staircase-like protein expression in most of the transcription units, and found high correlation abundances between some well-known complex subunits. In contrast, Schmidt et al. (2011) found only 5% “staircase behavior” for *L. interrogans* operons on the proteome level. These contradictory results reflected the cons of these label-free MS approaches: the technical variations and relatively low number of quantified proteins restricted the accurate and in-depth coverage of operon-controlled genes.

In this work, we set out to tackle these problems by employing a highly accurate label-free method, DIA (data-independent acquisition) (Purvine et al., 2003), to obtain quantification of the proteins constituting the *Escherichia coli* proteome with a high coverage and high accuracy. DIA is a MS-based proteomics method used in peptide quantification, in which all ions within a selected m/z range are fragmented and analyzed in a second stage of tandem mass spectrometry (Law and Lim, 2013). Although not suitable for discovery-based applications, DIA provides accurate peptide quantification without being limited to profiling predefined peptides of interest (Chapman et al., 2014; Doerr, 2015). This allowed us to investigate the protein abundances within operons and thus to interrogate the possible stoichiometry in operons of different functions.

## Materials and Methods

### MS Sample Preparation

*Escherichia coli* K-12 sub-strains BW25113 and MG1655 were cultivated on glucose M9 minimal medium at 37°C in flasks to mid-exponential phase (OD_600_ = 0.6) and then harvested in 45 mL volume, immediately cooled in ice water, and then centrifuged at 10,000 × *g* for 5 min. The pellet was washed once with PBS, centrifuged at 10,000 × *g* for 5 min again. Pellet was re-suspended on ice with lysis buffer (5 M urea/2 M thiourea in 10 mM HEPES, pH 8.0), and were sonicated and centrifuged at 17,000 × *g* for 30 min in a table-top centrifuge to remove cell debris. Supernatant was collected, and protein concentrations were determined with a Bradford Protein Assay (Bio-Rad Protein Assay Dye Reagent Concentrate, Cat. #500-0006).

For proteome analysis, we employed in-solution protein digestion with a filter-aided sample preparation (FASP) method (Zhang et al., 2017). 1 mg of protein was subjected to reduction (8 M urea and 50 mM DTT at 37°C, 1 h), followed by alkylation with 100 mM iodoacetamide (IAA) in dark at room temperature for 30 min. The solution was transferred to the 30 kDa ultracentrifuge filters (Millipore). Proteins were washed with 8 M urea, and four sequential buffer changes were performed using 50 mM TEAB, respectively. Trypsin (Promega) was then added into the filter at a mass ratio of 1:20 for Proteins digested in 130 μL 50 mM TEAB at 37°C for 12 h. The released peptides were collected by centrifugation and dried with a cold-trap speed vacuum.

### MS Experiments

One microgram of sample abovementioned peptides was analyzed on a C18 column (50 μm × 15 cm, 2 μm, Thermo Fisher) by using an EASY-nLC 1200 UHPLC connected to an Orbitrap Fusion Lumos mass spectrometer (Thermo Scientific). The peptides with the iRT-standard (1/10 by volume, Biognosys, HRM Calibration Kit: Ki-3003) were separated by a linear gradient from 6 to 30% ACN with 0.1% formic acid at 270 nL/min for 100–130 min and linearly increased to 90% ACN in 20 min. For the data-dependent acquisition (DDA), the source was operated at 2.0 kV. The DDA scheme included a full MS survey scan from *m/z* 400 to *m/z* 1500 at a resolution of 60,000 FWHM with AGC set to 4E5 (maximum injection time of 50 ms), followed by MS/MS scans at a resolution of 15,000 FWHM with AGC set to 5E4 (maximum injection time of 30 ms), data-dependent mode was set to top speed. Isolation window was 1.6. Dynamic exclusion was set to 90 s with a 10 ppm tolerance around the selected precursor. For the DIA hyper reaction monitoring (HRM-MS), individual tryptic peptide samples were mixed with the iRT-standard (1/10 by volume) and analyzed by the same method as DDA used. The method consisted of a full MS1 scan at a resolution of 60,000 FWHM from *m/z* 350 to *m/z* 1,200 with AGC set to 4E5 (maximum injection time of 30 ms) followed by 40 non-overlapping DIA windows acquired at a resolution of 30,000 FWHM with AGC set to 5E5 (maximum injection time of 50 ms), cycle time, 3.28 s. The MS/MS isolation windows were listed in Supplementary Table S1. For comparison, standard DDA MS experiment was performed as above. All MS raw data have been deposited in iProX with accession number: IPX0001095000 and ProteomeXchange with identifier PXD010126.

### Spectral Library Generation

To generate the spectral library, three DDA measurements of the mixed samples were performed. Raw DDA datasets were searched against a combined database of the NCBI database of *Escherichia coli* str. K-12 (GCF_000005845.2_ASM584v2, 4140 entries) and the iRT standard peptides sequence using the Sequest HT (Proteome Discoverer v2.1) local server. Common contaminants in the database included trypsin and keratins. Precursor and product ion spectra were searched at an initial mass tolerance of 10 ppm and fragment mass tolerance 0.02 Da, respectively. Tryptic cleavage was selected, and up to two missed cleavages were allowed. Carbamidomethylation on cysteine (+57.021 Da) was set as a fixed modification, and oxidation (+15.995 Da) on methionine was assigned as a variable modification. A target-decoy-based strategy was applied to control peptide and protein false discovery rates (FDRs) at lower than 1%. Confident protein identifications should suit the following criteria: (1) protein level FDR ≤ 1%; (2) unique peptides ≥ 1 or 2; (3) peptide length ≥ 6 or 7 aa. The search result was exported in a pdResult file format containing the annotation of precursors and fragment ions and their exact retention times. The pdResult file was then imported into Spectronaut Pulsar 11 (Biognosys) to generate the spectral library used for HRM-MS data analysis, which yielded 14608 unique peptide sequences in 2041 protein groups with BW25113, and 8822 unique peptide sequences in 1607 protein groups with MG1655. A subset of identified peptides was used in library creation as modification parameter was set none. The generated spectral libraries were exported from Spectronaut as in Supplementary Table S2.

### Protein Identification and Quantification

The DIA data were then analyzed with Spectronaut Pulsar 11 with the spectral library, which is a mass spectrometer vendor independent software for SWATH/DIA data analysis. Raw data were analyzed according to the user manual of the software. Default settings were setup for protein identification and peak area calculation. Raw data were converted into HTRMS files and imported to Spectronaut Pulsar 11 by choosing the matched database fasta file and spectral library, with the default settings of the Spectronaut Pulsar 11: (1) Calibration: calibration mode, automatic; iRT calibration strategy, non-liner iRT calibration. (2) Identification: decoy limit strategy, dynamic; decoy method, mutated; machine learning, per run; precursor q-value (peptide FDR) cutoff, 0.01; protein q-value (protein FDR) cutoff, 0.01; *p*-value estimator, kernel density estimator. (3) Workflow: default labeling type, label; profiling strategy, none; unify peptide peaks, false. (4) Quantification: interference correction, true; major(protein) grouping, by protein-group id; major group quantity, mean peptide quantity; minor (peptide) grouping, by stripped sequence; minor group quantity, mean precursor quantity; minor group top n, true; min, 1; max, 3; quantity MS-level, MS2; quantity type, area; data filtering, *q*-value; cross run normalization, true; row selection, *q*-value sparse; normalization strategy, local normalization. (5) Reporting: scoring histograms, true; pipeline report schema, protein quant; pipeline reporting unit, experiment. (6) Protein inference: protein inference workflow, automatic. (7) Data extraction: MS1 mass tolerance strategy, dynamic; correction factor, 1; MS2 mass tolerance strategy, dynamic; correction faction, 1. (8) Post analysis: differential abundance grouping, major group (quantification settings); smallest quantitative unit, precursor ion (summed fragment ions); use top n selection, false. (9) Retention time were used to assist identification. XIC extraction: XIC RT extraction window, dynamic; correction factor, 1. After peak extraction and area calculation were performed, the result was exported as the table format for further quantification analysis in Microsoft Excel. All MS raw data, Proteome Discoverer report (^∗^.msf file) and the constructed spectra library have been deposited in iProX with accession number: IPX0001095000 and ProteinXchange with identifier PXD010126. The relationship of submitted raw data are shown in Supplementary Data.

Protein abundances was calculated by using Spectronaut Pulsar protein pivot report, those proteins quantified by Spectronaut pulsar but not identified by Proteome Discoverer and not met the confident protein identifications criteria were removed. The abundances of identified proteins were calculated as follow procedure. Supposed that top 500 abundant proteins of *E. coli* can represent total protein copy numbers of a cell. Concentration of HRM-MS protein copies per cell was calculated based on the means of 500 most abundant protein quantities computed by other three label-free methods (APEX, iBAQ, PAI) downloaded from Arike et al. (2012). HRM-MS intensity could be converted to protein copies per cells by coefficient *k*, which is defined by the following formula.

*B*_i_ is copy numbers of the gene in iBAQ dataset.

*P*_i_ is copy numbers of the gene in emPAI dataset.

*A*_i_ is copy numbers of the gene in APEX dataset.

*D*_i_ is correspondence protein intensities of the gene quantified in HRM-MS dataset.

(1) $$k=\frac{\sqrt[{n}]{\prod_{i=0}^{n}\frac{\left(\mathrm{Bi}+\mathrm{Pi}+\mathrm{Ai}\right)}{3}}}{\sqrt[{n}]{\prod_{i=0}^{n}\mathrm{Di}}}$$

The amount of individual proteins was calculated as the product of conversion coefficient *k* to their intensity in the HRM-MS measured sample.

(2) Protein copy number=k×Di

The calculations were performed by in-house generated python scripts. All scripts used in this study can be downloaded in the Supplementary Materials (Supplementary Scripts).

### Coefficient of Variation of Protein Abundance in the Operons

Coefficient of variation (CV), which is defined as the ratio of the standard deviation to the arithmetic mean. Standard deviation is normalized by *n* − 1 by default (*n* is sample size). The CV of proteins within one operon is defined as the ratio of the standard deviation of protein quantities within this operon to the arithmetic mean of all protein quantities within this operon. For multi-gene operons (protein numbers ≥ 2), CV was calculate as follows:

(3) $$\mathrm{CV}=\frac{\sqrt[{2}]{\frac{{\sum_{i=1}^{n}\left(x_{i}-\bar{x}\right)}^{2}}{n-1}}}{\mathrm{mean}}\times 100\%$$

where *x_i_* is the abundance of the *i*-th gene in this operon. To be noted, the CV calculation was only performed within one operon, not across the operons.

The CV of the protein half-life in the operons were calculated in the same way. Protein half-life time in the M9 minimal medium was from our previous work (Zhong et al., 2015).

### Data Randomization

To compare with the real operon CV level if protein abundances in operons have stoichiometry control, we reshuffled the protein quantities detected in the polycistronic operons (846) randomly to each protein ID, the generated dataset was used as randomized negative control. Randomized protein quantities in “2-/3-/4-/≥5-protein” operons were extracted from this randomized negative control data.

### Operon and Gene Ontology (GO) Analysis

Operon library of *Escherichia coli* str. K-12 were downloaded from the DOOR^2^ database (Mao et al., 2014) (NC_000913). Protein GI numbers were converted to proper identifiers by DAVID Gene Accession Conversion Tool (Huang et al., 2007). The quantified proteins were integrated to the operon data. The PANTHER Version 13.0 (released 2017-11-12^1^) (Mi et al., 2017) was used to perform the GO overrepresentation analysis with the significance threshold of 0.01, the quantified proteins of *Escherichia coli* in our work was selected as the background proteome, the Fisher’s Exact test was used to obtain *p*-values and ‘GO slim’ category were used. The protein subcellular localization data of *E. coli* was downloaded from EcoProDB (Yun et al., 2007).

### Complex and Pathway Classification

The operon contains more than or equal to two genes were called polycistronic operons. Among polycistronic operons, those ≥90% genes in operon encoded subunits of one protein complex is selected and classified as “Complex” group, others were classified as “Pathway” group.

### Physical and Chemical Features of Proteins

The protein lengths in amino acids were obtained from the NCBI of *Escherichia coli* str. K-12 (GCF_000005845.2_ASM584v2, 4140 entries). Information of the hydrophobicity was calculated by Gravy Calculator^2^. In addition, the isoelectric point, protein length, instability and hydrophobicity distribution were calculated by using python 2.7 scripts and Biopython libraries.

### Experimental Design and Statistical Rationale

To increases the precision of protein expression measurements of the entire *E. coli* proteome quantification, two biological replicates of BW25113 and MG1655 each were cultured in M9 minimal medium to mid-exponential phase and were harvest, then processed to HRM-MS analysis independently. iRT-standard (Biognosys, HRM Calibration Kit) was added to the peptides with 1/10 by volume. The peptide mixture of two biological replicates of each strain were used and performed LC-MS for three times for spectral library creation. Proteome Discoverer 2.1 and Spectronaut Pulsar 11 were used to generated spectral library, and Spectronaut Pulsar 11 was used to quantify the protein groups with little modified parameters. Kolmogorov–Smirnov test (KS-test) were used to compare the distributions between CVs at transcriptome, translatome and protein level, and Mann-Whitney U-test were used to compare the difference between complex and pathway operons.

### mRNA Sequencing

*E. coli* strain BW25113 was cultivated on glucose M9 minimal medium at 37°C in flasks to mid-exponential phase (OD_600_ = 0.6) with 100 μg/mL chloramphenicol added 15 min before harvest, then the cells were centrifuged at 5,000 × *g* for 10 min at 4°C, followed by thrice washed with pre-chilled PBS. Cell pellet was then re-suspended in 6 mL pre-chilled sucrose-buffer solution [16 mM Tris (pH 8.1) supplemented with 0.5 M RNase-free sucrose, 50 mM KCl, 8.75 mM EDTA, 100 μg/mL chloramphenicol, 12.5 mg/mL lysozyme] and gently stirred for 5 min on ice. Then the cells were centrifuged 5,000 × *g*, 10 min. Total RNA was extracted by Trizol method, and mRNA-seq libraries were prepared using standard MGIEasy^TM^ mRNA Library Prep Kit V2 following the manufacturer’s protocol. Sequencing was performed on a BGISEQ-500 sequencer for 50 cycles, single-ended mode. This dataset was deposited in the GEO database under the accession number GSM3489376, GSM3489377.

### Analysis of Sequencing Data

The RNA-seq dataset of *E. coli* strain MG1655 were obtained from Haft et al. (2014) (GEO accession number: GSM1360030, GSM1360031, GSM1360042, GSM1360043) and Bartholomaus et al. (2016) (SRA accession number: SRR2016457). The datasets of strain BW25113 were generated as described above. For all datasets, adapters was trimmed from the reads. Reads were mapped to coding sequence of *E. coli* reference genome (GenBank: U00096) using FANSe3 algorithm (Liu et al., 2017) with the parameters -E3 -S10 –indel. Genes with at least 10 mapped reads were considered quantifiable (Bloom et al., 2009). The expression levels were estimated in rpkM.

## Results

### Near-Complete *E. coli* Cytosolic Proteome Quantification Using HRM-MS

**FIGURE 1:**
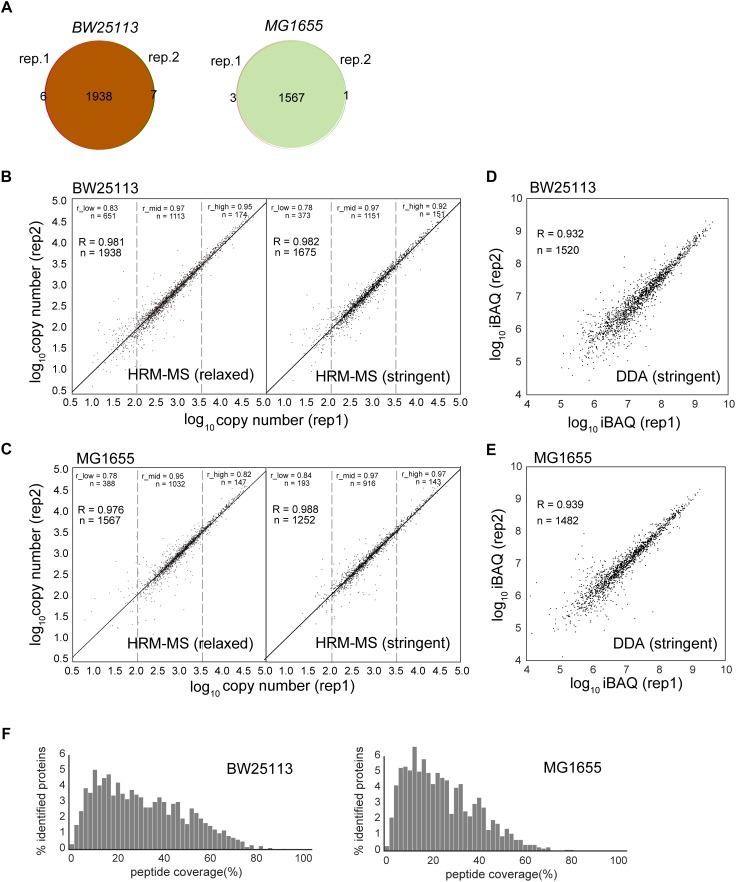
Comparison of protein abundances obtained by different label-free quantification methods. **(A)** Quantified protein numbers in two biological replications of HRM-MS method of two strains. **(B,C)** The quantification reproducibility of HRM-MS method in relaxed and stringent criteria of two *E. coli* strains, respectively. R is the Pearson correlation coefficient. The protein abundance range was divided into low (<2.0 log scale), mid (2.0–3.5 log scale) and high (>3.5 log scale) sections. r_low, r_mid and r_high are the Pearson correlation of the proteins in these sections. **(D,E)** The reproducibility of DDA MS experiment of the two strains. Proteins were quantified using iBAQ method. **(F)** The peptide coverage of the HRM-MS-identified proteins in two *E. coli* strains, respectively.

To assess the quantification power and reproducibility of the HRM-MS method, we performed two biological replicates of *E. coli* total soluble proteins of strain BW25113 and MG1655. When using the previous identification criteria (at least one unique peptide, peptide length ≥6 amino acids) (Arike et al., 2012), our HRM-MS results quantified 1951 and 1571 proteins in these two strains, respectively. The two biological replications identified almost identical proteins, demonstrating high robustness and reproducibility (Figure 1A). Two replicates quantified almost the same proteins: only a few proteins were quantified only in one replicate (Figure 1A). Under the stringent criteria as two unique peptides and at least seven amino acids peptide length. Even under the stringent criteria, we still quantified 1675 and 1252 proteins with a high reproducibility (Figure 1B,C and Supplementary Table S3). The number of quantified soluble proteins was almost doubled when compared to the previous results quantified by other methods (1021 proteins for APEX, 1183 for IBAQ and 1138 for emPAI) (Arike et al., 2012). To rule out the difference of the instruments, we performed DDA MS experiments for the two strains in the same Orbitrap Fusion Lumos instrument. Proteins were identified under the stringent criteria and quantified using iBAQ method. The DDA MS quantified 1520 and 1482 proteins for BW25113 and MG1655 strains, respectively, comparable with the HRM-MS experiments. However, the correlation coefficients of the iBAQ quantification of two biological replicates were 0.932 and 0.939, respectively (Figure 1D,E), lower than the HRM-MS (*R* = 0.982 and 0.988 for the two strains, respectively). Each identified protein was covered by 19.20 and 15.45 peptides in average in two strains, covering 31.56% and 24.87% of the amino acid sequences, respectively (Figure 1F), which is higher than the typical peptide coverage of human proteome MS experiments (single search engine, up to ∼20% coverage) (Zhao et al., 2017).

We previously revealed 2922 genes which are being translated in the *E. coli* grown in the same condition using ribosome profiling (Bartholomaus et al., 2016). Using HRM-MS method with stringent criteria, we quantified 55.2% of these translating genes in this work. We next analyzed the possible chemical and physical features of the translated but unquantified proteins in this work. The unquantified proteins are significantly more alkalic, less stable, shorter and more hydrophobic (Supplementary Figure S1). These are general factors that decreases the visibility of these proteins in shotgun MS experiments. Since our experiments were not optimized for membrane proteins, which are more prone to aggregate during the protein extraction, these proteins are expected to be less detected in the MS. Notably, we quantified 93.6% translating cytosolic proteins, showing a near-complete quantification of the soluble proteins.

Considering the advantage of the HRM-MS, all the subsequent analysis was based on the HRM-MS under the stringent criteria.

### Operons Tend to Unify Gene Expression in General

**FIGURE 2:**
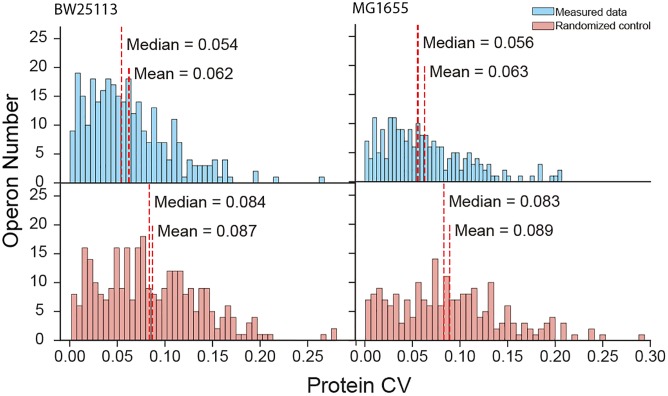
Protein coefficient of variation (CV) within operons of measured data and randomized negative control.

The high coverage of proteome quantification allowed us to make an in-depth investigation of protein abundances in operons. Indeed, our quantification covered 67.9% and 56.0% of the translating polycistronic operons in BW25113 and MG1655, respectively. We calculated the coefficient of variation (CV, %) of proteins abundances within each operon. To compare with the real operon CV level, we random redistribution the protein quantities quantified in the experiment as randomized negative control. Smaller CV represent the unified expression level of the proteins within one operon. The mean of real CV was significantly smaller than that of randomized negative control data (Mann–Whitney *U*-test, *p* = 5.05 × 10^−10^ and *p* = 3.40 × 10^−5^ for BW25113 and MG1655, respectively) (Figure 2). The median CV of quantified proteins were also smaller than the randomized control (Kruskal–Wallis H-test, *p* = 1.44 × 10^−8^ and *p* = 1.18 × 10^−6^ for BW25113 and MG1655, respectively). As a positive control, the trend to unified expression is also valid in transcription (an operon is transcribed as an entire mRNA) (Supplementary Figure S2). This indicated that most polycistronic operons were co-regulated, and the stoichiometry balance of protein abundances within polycistronic operons exists in general, although with exceptions. These results were in general consistent with previous studies (Ishihama et al., 2008; Arike et al., 2012).

### Functional Enrichment of the Operon Stoichiometry Control

**FIGURE 3:**
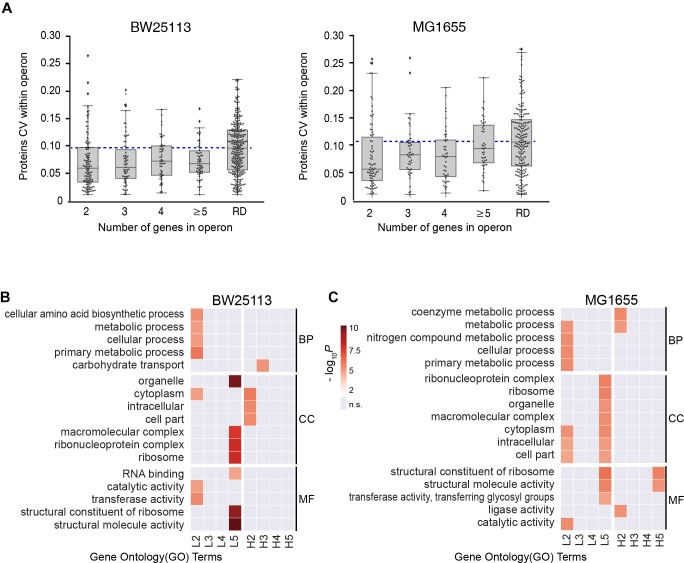
Functional-dependence of operon stoichiometry control. **(A)** Protein CVs within operons, categorized according to the number of genes in operon. RD, randomized data. Blue dashed line represents the median of the randomized data. **(B,C)** Gene ontology (GO) overrepresentation of the operons with lower CV than the randomized median (L2–L5) and higher CV than the randomized median (H2–H5). The number refer to the number of genes in operon. GO terms with *P* > 0.001 were considered insignificant and marked as gray. BP, biological process; CC, cell component; MF, molecular function. **(B)** BW25113 and **(C)** MG1655 strain.

We then examined if the number of genes per operon could affect the stoichiometry of proteins encoded within operons in both BW25113 and MG1655 strains. We found all the medians of subgroups were lower than the randomized negative control (RD), which was in accordance with our abovementioned results (Figure 3A), indicating operon expression regulation exists in general. We continue to divide operons into two subgroups by their functions.

Next, each group was separated into two subgroups by the median CV of randomized data, the high CV subgroups (H2-H5, higher than median of randomized data) and the low CV subgroups (L2–L5, lower than median of randomized data). Gene ontology (GO) overrepresentation analysis was performed for each subgroup both in both strains (Figure 3B,C, see details in Supplementary Table S5). Most low CV subgroups (L2–L5) showed functional enrichment against the quantified proteins as background. The L2 group was overrepresented in almost all metabolic activities, and the L5 group was highly enriched in complexes and structural molecules in both *E. coli* strains. This provided a hint that the stringent stoichiometry control might be important for the efficient assembly of protein complexes. In contrast, there are little significant functional enrichment of GO terms enriched in H2–H5 subgroups in BW25113, and only the GO terms enriched in H2 and H5 subgroup in MG1655. These results indicated that the large CV of most of these operons might be caused by experimental error.

Since the operons in L2–L5 subgroups were enriched in metabolic pathways and complexes, we specifically divide these operons into “Complex” and “Pathway” groups. The group “Complex” are the operons encoding proteins for the same protein complex. The group “Pathway” are the operons encoding proteins involving in the same metabolic pathway. Similar to the Figure 3A, we performed randomization for each group multiple times for robustness. In almost all cases the 2-/3-/4-/≥5-protein “Complex” and “Pathway” operons exhibited lower CVs than the corresponding randomized data (Supplementary Figure S3), indicating that the stoichiometry is still maintained for the protein complexes and metabolic pathways in a certain extent, which is consistent with the traditional hypothesis.

However, there seems to be a trend that shorter operons (containing lower number of genes) possess lower CV within operon (Figure 3A), suggesting a length-dependent stoichiometry control. Since the mRNA of an entire operon is transcribed as a unit, the abovementioned phenomenon opened a question of the origin of the length-dependent stoichiometry control.

### Length-Dependence of Stoichiometry Control Is Functional-Dependent

**FIGURE 4:**
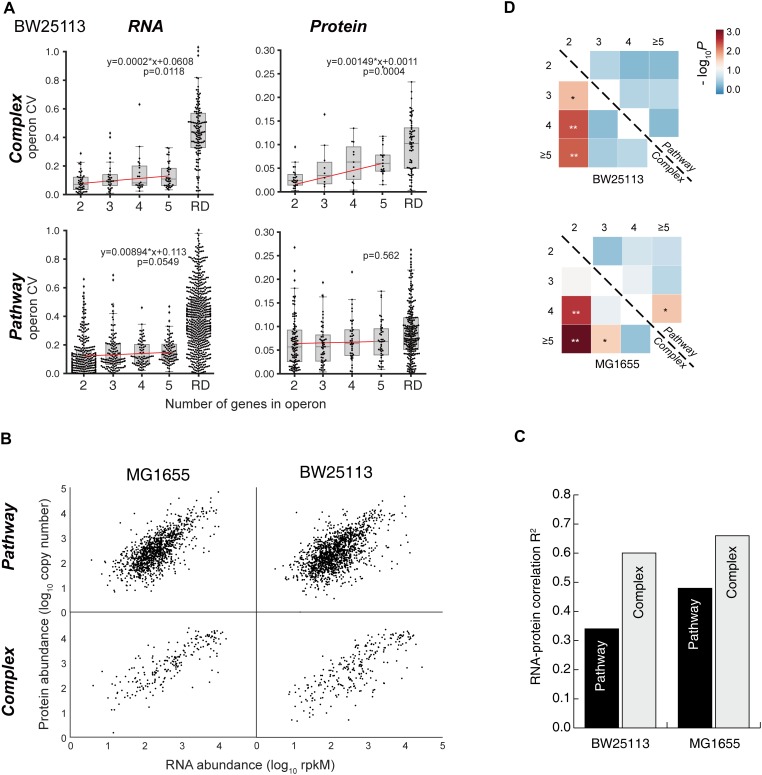
Length-dependence of operon stoichiometry control. **(A)** Linear regression analysis of gene CVs at RNA and protein levels within operon that encodes proteins forming complexes or involving in the metabolic pathways in BW25113 strain. *P*-values of the regression are indicated in the plots. *P* < 0.05 are considered significant. RD, randomized control. **(B)** The RNA–protein scatter plots of the gene expression levels of the “Complex” and “Pathway” operons in two strains, respectively. **(C)** The correlation coefficient *R*^2^ of the RNA–protein correlation shown in **(B)** panel. **(D)** The mutual *P*-value (Mann–Whitney *U* test) matrix of the protein CV within “Pathway” operons and “Complex” operons, respectively. ^∗^*P* < 0.05; ^∗∗^*P* < 0.01.

Linear regression analysis was performed to calculate the CVs at RNA and protein levels, within operon that encodes proteins forming complexes or involving in the metabolic pathways (Figure 4A and Supplementary Figure S4). Since the mRNA of one operon is transcribed as one unit, the CV within operon at RNA level is much lower than the randomized control, as expected (Figure 4A and Supplementary Figure S4). However, the “Pathway” operons did not show length dependence at protein level, while significant and positive correlation of protein CV within operon versus length were observed in “Complex” groups in both BW25113 and MG1655 (*P-*value of BW25113 = 0.0004, *P-*value of MG1655 = 0.022). In contrast, among the “Pathway” groups, those operons exhibited similar CV distribution regardless of their lengths (regression *P* > 0.05) (Figure 4A, “Protein” plots). This reflects necessity that the proteins operating in a pathway need to be more independently tuned and thus do not have to follow the stoichiometry. Those results indicating a length- and functional-dependence of operon stoichiometry control for larger operons. The RNA–protein correlation also echoed this trend (Figure 4B). In both strains, the Pearson *R*^2^ of the RNA–protein correlation in “Complex” group is considerably higher than in “Pathway” group (Figure 4C). These results indicated that there may be some inherent difference between “Pathway” and “Complex” type operons.

Significant difference (*p* < 0.01) of protein CV distribution were observed among the 2-protein operons against larger (4-/5-protein) operons only in “Complex” subgroups, but not observed in the “Pathway” operon subgroups in both BW25113 and MG1655 strains (Figure 4D, see details in Supplementary Table S4), consolidated our abovementioned observation that the shorter operons among “Complex” groups operons tend to be regulated more stringently.

### Enzyme Activity Correlates to the Differential Translation of “Pathway” Genes

**FIGURE 5:**
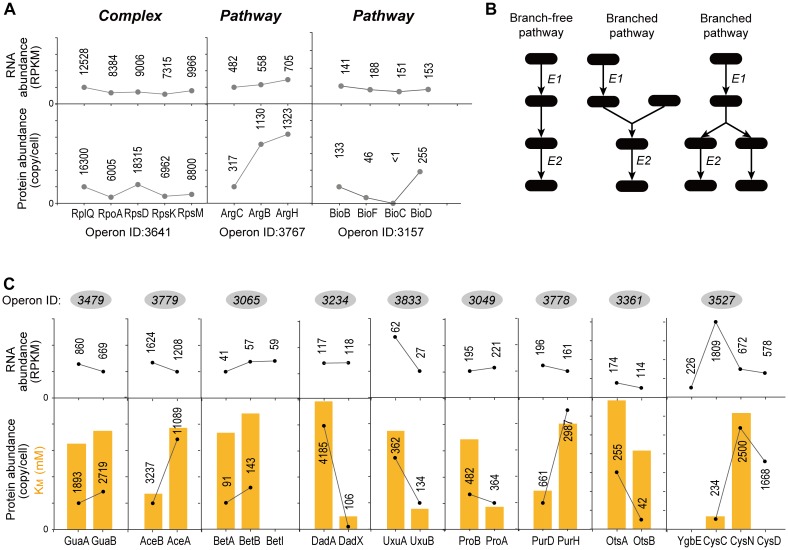
Examples of “Complex” and “Pathway” operons. **(A)** The gene expression at RNA and protein levels in *E. coli* K-12 MG1655 strain. The detailed expression level are marked on the data points. <1 means the expression level is too low to be confidently quantified. **(B)** Illustrations of the “branch-free” and “branched” pathways. E1 and E2 represent the quantified proteins in the same operon. **(C)** All nine operons which encodes enzymes that exist in branch-free pathways. Their RNA abundance, protein abundance and *K*_M_ values were plotted. *K*_M_ values are plotted in orange bars. Detailed *K*_M_ values are listed in Table 1. Detailed pathways are illustrated in Supplementary Figure S6.

Figure 5A showed examples of “Complex” and “Pathway” operons, respectively. Genes in three operons showed almost same RNA abundance. However, the operon ID 3641 encoding ribosome proteins showed similar protein abundance, while the operon ID 3767 encoding enzymes in arginine synthesis pathway and the operon ID 3157 encoding enzymes in biotin synthesis pathway showed exaggerated difference in protein abundance. Both translation and degradation may affect the protein abundance. We found no significant difference of the “Complex” and “Pathway” operons in terms of the CV of protein half-life within operons (KS-test, *P* = 0.932) (Supplementary Figure S5). Therefore, the translational regulation should be the major factor leading to such differential protein expression in “Pathway” operons.

It is expected that the “Complex” proteins tend to be tightly stoichiometrically controlled to build functional complexes. In contrast, what benefit is related to the differential translation of the “Pathway” genes in one operon?

Since “Pathway” proteins comprise pathways, they sequentially catalyze conversion of a substrate to product via multiple and successive reaction steps. Therefore, we hypothesize that the bacteria require less high-efficiency enzymes to avoid energy waste. Supplementary Figure S6 showed the arginine synthesis pathway in *E. coli*, where ArgB and ArgC are two enzymes that relay. The Michalis constant (*K*_M_) of ArgB is more than 3 times higher than ArgC (Table 1), showing that the binding of ArgB to the substrate is weaker. Although lacking the measured value of ArgB *k*_cat_ in *E. coli*, ArgB *k*_cat_ value in yeast (56% homology in amino acid sequence) is approximately 1/3 of ArgC in *E. coli*, as a reference. These data indicated that the catalytic efficiency of ArgC is higher than ArgB. Therefore, ArgC is less needed in *E. coli*, which matched the proteome quantification (Figure 5A). No argH activity data is available in *E. coli*. The homologous enzyme in *Anas platyrhynchos* (75% homology in amino acid sequence) showed also lower catalytic efficiency than ArgC. Similar trend was also observed in the biotin synthesis pathway (Supplementary Figure S6). The enzyme BioF possess *k*_cat_ value one order of magnitude higher than BioB, indicating higher conversion efficiency. Therefore, BioF is much less produced in *E. coli*. The enzyme BioC is extremely efficient with three orders of magnitude higher *k*_cat_ value than BioF and much lower *K*_M_ value than bioB and BioF. Therefore, BioC is orders of magnitude lower than the other two enzymes in *E. coli*.

**Table 1:** Kinetic parameters of the enzymes in one metabolic pathway.

Enzyme	*k*_cat_ (s^−1^)	*K*_M_ (mM)	Homology to *E. coli* protein (for non-*E. coli* proteins)	References
ArgB	4.9 (*S. cerevisiae*)	1.3	56%	Gil-Ortiz et al., 2010; de Cima et al., 2012
ArgC	14	0.4		McLoughlin and Copley, 2008
ArgH	4.9 (*Anas platyrhynchos*)	0.4 (*Anas platyrhynchos*)	75%	Chakraborty et al., 1999
BioB	0.0039	0.002		Farh et al., 2001; Taylor et al., 2008
BioF	0.05825	0.025		Turbeville et al., 2011
BioC	98.334 (*Bacillus cereus*)	0.00108 (*Bacillus cereus*)	62%	Lin and Cronan, 2012
GuaB	13	0.061		Kerr and Hedstrom, 1997
GuaA	23	0.053		Oliver et al., 2014
AceB		0.022		Lohman et al., 2008
AceA		0.063		MacKintosh and Nimmo, 1988
BetA		1.5		Landfald and Strom, 1986
BetB		1.8		Gruez et al., 2004
UxuA		4.79		Qiu et al., 2012
UxuB		1		Hickman and Ashwell, 1960
DadA		30		Franklin and Venables, 1976
DadX		3.03		Wu et al., 2008
ProB		1.2 (*Pseudomonas aeruginosa*)	74%	Krishna and Leisinger, 1979
ProA		300		McLoughlin and Copley, 2008
OtsA		1 (*Thermoplasma acidophilum*)	65%	Gao et al., 2014
OtsB		0.61		Kuznetsova et al., 2006
CysC		0.0005		Satishchandran and Markham, 2000
CysD + CysN		0.0045 (*Thiobacillus denitrificans*)	67% (CysD)	Gay et al., 2009
PurD		0.03		Cheng et al., 1990
PurH		0.082 (*Methanocaldococcus jannaschii*)	50%	Graupner et al., 2002

*Values are measured in E. coli unless specified. Non-E. coli data were used as a reference when no E. coli data is available.*

To further validate this hypothesis, we manually went through all quantified “Pathway” operons and found other nine operons which fit the following criteria: (a) at least two quantified proteins in one operons, and they must exist in the same metabolic pathway; (b) their pathway must be branch-free, i.e., the substrate should be converted sequentially by these enzymes without introducing other rate-limiting metabolites as branches in pathway, at least in the range of the quantified enzymes (illustrations see Figure 5B); (c) enzyme activity parameters, e.g., *k*_cat_ or *K*_M_, should be available, and the activity parameters of at least one enzyme should be available in *E. coli*. The RNA abundance, protein abundance and the *K*_M_ values are illustrated in Figure 5C. All of the nine operons validated the correlation of the enzyme activity and the protein abundance without exception: less active enzymes (represented by higher *K*_M_ values) were expressed in higher amount at protein level. To be noted, six out of nine operons showed inverse proportion of RNA and protein expression, suggesting that such regulation is mainly conducted at translation level.

### Divergence of Stoichiometry Control Is Regulated at Translation Level by the Gene Intervals

**FIGURE 6:**
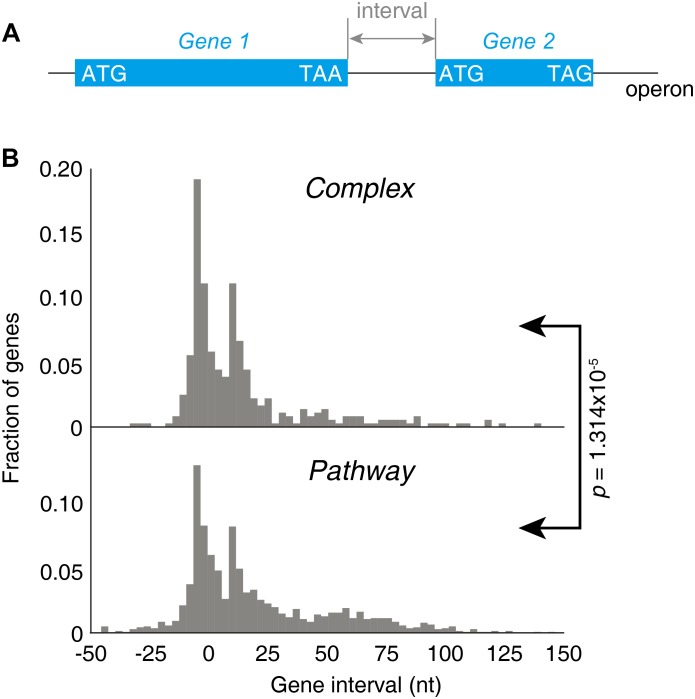
Gene intervals in the “Complex” and “Pathway” operons. **(A)** Illustration of the gene interval within one operon. **(B)** Distribution of the gene intervals of “Complex” and “Pathway” operons.

Next, we investigated the factor that could determine the lower stringency of “Pathway” groups of operons. As both groups of operons contain a wide variety of genes, the major difference should lay on the gene structures. This is reflected by the gene intervals, defined as the distance from the stop codon of the first gene to the start codon of the next gene downstream within one operon (Figure 6A). A strongly significant difference on gene intervals was observed between two groups on their gene interval distributions (*p* = 1.314 × 10^−5^, KS-test, Figure 6B). The genes in “Complex” genes tend to be arranged very near to each other, while the “Pathway” genes tend to be located far from each other, reflected by the larger mean and median value of the gene interval.

Both a 70S ribosome and a 30S subunit cover about 40 nucleotides of the mRNA, roughly 18–20 nucleotides upstream and 16–18 nucleotides downstream of the P-site codon (Beyer et al., 1994). If a terminating 70S ribosome would have a downstream initiation site nearer than 40 nucleotides, a 70S termination event and a downstream 30S-binding initiation could not be independent events due to a steric clash between 70S and 30S. In this case, the 70S scanning initiation mode plays a major role for translation, meaning the first cistron is initiated by 30S and the downstream cistrons by 70S scanning to achieve a strict and precise 1:1 stoichiometric ratio (Yamamoto et al., 2016). This may be one of the factors explained that the “Complex” operons, with shorter gene intervals, are more stringently regulated for stoichiometry.

## Discussion

Near-complete coverage of proteome identification and quantification is always a goal of proteomics research, as it reveals detailed global dynamics of important biological processes; for example, the dormancy and resuscitation of *Mycobacterium tuberculosis* (Schubert et al., 2015). The high proteomic resolution of quantification allows in-depth investigation of many scientific questions in debate for decades. In this study, we employed DIA based HRM-MS, a highly reproducible label-free quantification method in *E. coli* strains BW25113 and MG1655. Our datasets were better than the previously reported DDA-based *E. coli* proteome quantification datasets in terms of reproducibility. We therefore generated the most complete investigation on abundance of proteins encoded within operons in *E. coli* based on tryptic digestion up to date, which allows us to accurately evaluate the stoichiometry presumption of the operon organization. To further increase the sensitivity of identification, other complement approaches such as LysC digestion might be helpful (Wisniewski and Rakus, 2014). Nevertheless, since we have already quantified 93.6% of the cytosolic proteins, specific methods dealing with the hydrophobic nature of membrane proteins should be employed to further expand the proteome coverage. The DIA based HRM-MS relies heavily on DDA spectral libraries. Therefore, they share the same shortcomings, e.g., the dependence on the physical and chemical properties of proteins.

We confirmed from our HRM-MS results that the operons coordinate the gene expression more stringent than the randomized control in general. In addition, we found a multifaceted nature of the operon regulation: operons are not created equal. The stringency is length-dependent and functional-dependent at protein level. Such multifaceted organization of operons revealed two-level control: the operon unified transcriptional level and gene-specific translational level, which benefits the host in different aspects.

Although the operon organization maintains in general the stoichiometry of the genes in the operons compared to fully randomized scenario, the operons for metabolic pathways are in general less strictly controlled for stoichiometry balance compared to those operons for protein complexes. Protein complexes needs stoichiometry to maintain their functions. Therefore, the operons encoding protein complexes are tightly regulated to ensure the equal expression, such as the ribosome protein operons (Figure 5A). In contrast, since the operons for metabolic pathways are not necessarily forming a complex for their functions, their distinct specific activities set their specific demand in quantity. All quantified operons which encode enzymes in branch-free pathways and with available enzyme activity data validated this hypothesis without exception (Figure 5C). Nevertheless, bacteria need to regulate related metabolic pathways in quick response to environmental stimuli. Therefore, organizing the proteins in the same pathway under the control of one promoter would minimize the regulatory complexity of the adjustments, leaving the delicate control of each individual gene to the translational level. The available data indicated that such translational regulation is quite common (Figure 5C).

Our data also showed that the shorter operons, whose products form complexes, tend to be more tightly controlled in stoichiometric expression (Figure 4). This could be understandable such as two-component protein complexes would be invalid if there were imbalanced expression of the components. For instance, the assembly efficiency decreased remarkably if two subunits of bacterial luciferase *LuxA* and *LuxB* were split at distant chromosomal sites (Shieh et al., 2015). Large operons tend to encode proteins for large complexes such as ribosomes (Figure 4). Such large complexes would sustain for relatively long time in cells to perform essential functions. For example, half-life of mammalian ribosomes can be as long as 300 h (Nikolov et al., 1983). Many protein components of ribosomes are dissociable and interchangeable with unbound counterparts (Schafer et al., 2003). This allows the rapid renewal of the damaged proteins of the complex without degrading and re-synthesizing the entire complex, which is the most energy-efficient way to keep these valuable complexes in good condition. This requires the delicate expression control of these proteins within one operon to meet the actual demand.

In another aspect, the differential expression regulation within an operon is also important for bacteria. Previous studies proposed that such regulation happens via generating different transcripts from multiple promotors/terminators [e.g., *Bacillus subtilis dnaK* operon (Homuth et al., 1999), *Vibrio vulnificus putAP* operon (Lee et al., 2003), *Zymomonas mobilis gap* operon (Eddy et al., 1989), *E. coli glpEGR* operon (Yang and Larson, 1998)], or via differential degrading mRNAs [e.g., *Acinetobacter calcoaceticus mop* operon (Schirmer and Hillen, 1998), *E. coli atp* operon (McCarthy, 1990; McCarthy et al., 1991)]. However, these studies included only individual cases of specific operons. Taking advantage of deep coverage of *E. coli* cytosolic proteome, our data indicated that enzyme activity seems to be an additional driving force for the differential expression regulation within an operon. Highly efficient enzymes tended to be less produced than the other counterparts in the same pathway. In such cases, deviating from stoichiometry minimizes the energy waste and thus may provide survival advantage. This explained the fact that no significant length-dependent stoichiometry is observed in “Pathway” proteins. Our analysis was restricted by the very limited availability of the enzyme activity and kinetics data. Validation using more such data is necessary in the future.

In this study, we noticed that the gene intervals in operons may serve as a regulatory factor for stoichiometry. It is a general accepted notion that termination of bacterial protein synthesis is obligatorily followed by recycling step governed by the factors ribosomal recycling factor (RRF), EF-G, and IF3, where the ribosome dissociates into its subunits (Hirokawa et al., 2006). In contrast, a recently described 70S-scanning mode of initiation holds that after termination, the 70S ribosomes do not dissociate after termination step but rather scan along with the mRNA until reaching the initiation site of the downstream cistron of the same mRNA (Yamamoto et al., 2016). Binding of fMet-tRNA triggers 70S scanning, which occurs in the absence of energy-rich compounds (e.g., ATP, GTP) and seems to be driven by unidimensional diffusion (Yamamoto et al., 2016). Therefore, the 70S scanning initiation might be a mechanism to read out the “stoichiometry code” of closely located genes in operons. In addition, the rate of translation of an ORF is controlled by a number of other mRNA features. For example, the codon selection and the cognate tRNA concentration dictate the translational pausing, which is a strong determinant of co-translational folding for most proteins (Zhang et al., 2009; Zhong et al., 2015; Lian et al., 2016). Shine–Dalgarno (SD) sequence accessibility and strength have been implicated in translational initiation (Steitz and Jakes, 1975). Genome-wide mRNA secondary structure analysis indicated that ORF translation rate is correlated with its mRNA structure in bacteria (Burkhardt et al., 2017), but not in mammalian cells (Lian et al., 2016). Although highly stable mRNA structures in direct proximity to the initiation codon diminish translation efficiency (de Smit and van Duin, 1990), secondary structure hiding the SD sequence in front of the second cistron prevents 30S binding initiation, but the secondary structure can be resolved by scanning 70S ribosomes when the secondary structure has a comparable stability (ΔG ≥ −6 kcal/mol at 30°C) (Qin et al., 2016; Yamamoto et al., 2016). These molecular mechanisms should be universal beyond exponential growth condition, although further validation would be needed.

This two-level regulation mode involving transcription and translation would balance the regulation in different time-scale. As transcriptional control takes effects at least in half an hour, it is suitable for sustained alteration in gene expression and in pathway-scale. In contrast, rapid and fine adjustment can be only performed at the translational level, which takes effects in less than 1 min and occurs at the individual gene level (Zhong et al., 2015). This rapid responsiveness would be also ideal for real-time adjustment of the proteins needed in complexes and pathways. Translational regulation largely contributes to the proteome complexity and minimizes the energy waste on synthesizing unnecessary proteins. Thus, the delicate and differential translational regulation in bacteria maintains the functionality and efficiency of both macromolecular complexes and metabolic pathways, which is a desperate need of the survival and competence of bacteria.

## Data Availability

The datasets generated for this study can be found in GEO, GSM3489376, GSM3489377.

## Author Contributions

GZ, JZ, and CS: conceptualization. JZ and HZ: experiment and visualization. JZ, HZ, BQ, and RN: data analysis. GZ and Q-YH: supervision. JZ, HZ, BQ, RN, Q-YH, CS, and GZ: writing. GZ: funding acquisition.

## Conflict of Interest Statement

The authors declare that the research was conducted in the absence of any commercial or financial relationships that could be construed as a potential conflict of interest.

## Footnotes

1 http://pantherdb.org/

2 http://www.gravy-calculator.de

## Supplementary Material

FIGURE S1 Physical and chemical features of quantified and unquantified proteins in *E. coli*. Panels **(A–D)** are distributions of protein isoelectric point, instability, protein length, and hydrophobicity. Instability tests a protein for stability, which value above 40 means the protein has a shorter half-life. The Kolmogorov–Smirnov test (KS-test) was used to test the distribution between quantified and unquantified proteins. Significance different were found between quantified and unquantified proteins indicated different physical–chemical characterizations of them.

FIGURE S2 CV within operons of measured data and randomized negative control at RNA level.

FIGURE S3 Length- and functional-dependence of operon stoichiometry control compare to randomized data. **(A–E)** Distribution among “Complex,” “Complex_random,” “Pathway,” and “Pathway_random” groups in BW25113. **(F–J)** Distribution among “Complex,” “Complex_random,” “Pathway,” “Pathway_random” groups in MG1655. In most cases, the CV in “Complex” and “Pathway” groups were lower than corresponding “Complex_random” and “Pathway_random” groups in both strains, indicating real robust signals but not statistical artifacts in operons present in *E. coli.*

FIGURE S4 Length dependence of the stoichiometry control at RNA and protein levels. Same figure in MG1655 strain, comparable to the Figure 4A.

FIGURE S5 Distribution of protein half-life CV in “Complex” and “Pathway” operons.

FIGURE S6 Pathways in Figure 5.

TABLE S1 DIA MS/MS isolation windows table.

TABLE S2 Spectral library used in HRM-MS analysis.

TABLE S3 Protein abundances and properties used in this analysis.

TABLE S4 *P*-value of Mann–Whitney *U* test between complex and pathway operons shown in Figure 3C.

TABLE S5 *P*-value of gene ontology (GO) overrepresentation of the operons with lower and higher CV groups shown in Figure 3D,E.

SUPPLEMENTARY SCRIPTS The python scripts used in this study.

SUPPLEMENTARY DATA The relationship of submitted raw data.
