# Supplementary material for: Multifaceted Stoichiometry Control of Bacterial Operons Revealed by Deep Proteome Quantification
Source: Front Genet. 2019 May 24;10:473. doi: 10.3389/fgene.2019.00473 (PMC6544118; doi:10.3389/fgene.2019.00473)

Supplementary Material

Multifaceted stoichiometry control of bacterial operons revealed by deep proteome quantification

Jing Zhao* , Hong Zhang* , Bo Qin , Rainer Nikolay , Qing-Yu He , Christian M.T. Spahn *, Gong Zhang

*** Correspondence: Gong Zhang**: zhanggong-uni@qq.com

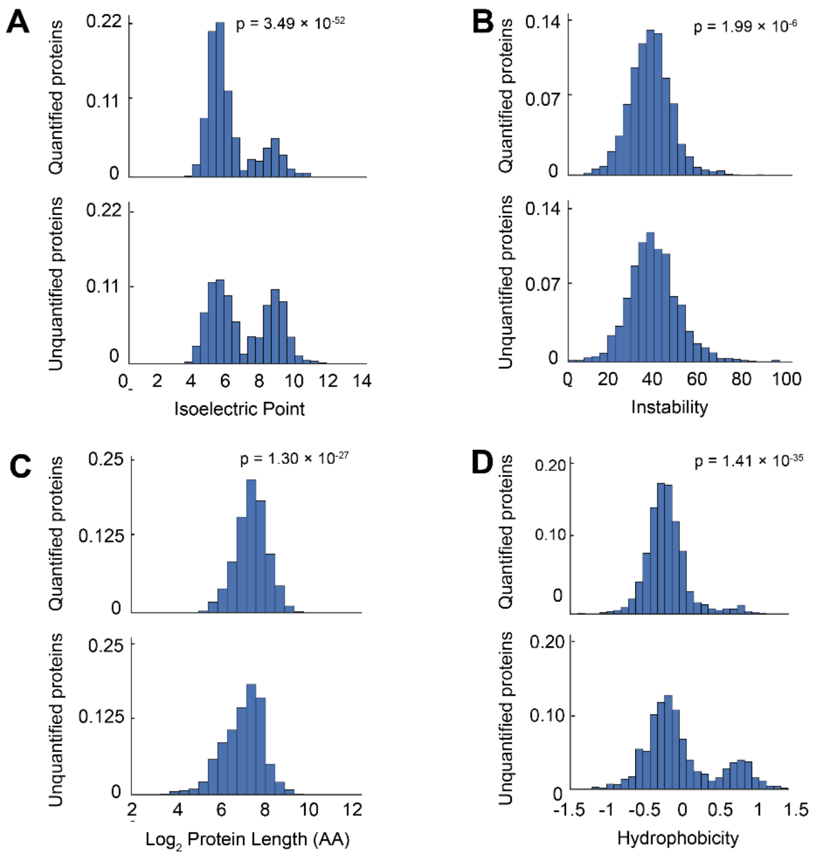

Supplementary Figure S1. Physical and chemical features of quantified and unquantified proteins in *E. coli.* (A-D) are distributions of protein isoelectric point, instability, protein length, and hydrophobicity. Instability tests a protein for stability, which value above 40 means the protein has a short half-life [1]. The Kolmogorov−Smirnov test (KS-test) was used to test the distribution between quantified and unquantified proteins. Significance different were found between quantified and unquantified proteins indicated different physical−chemical characterizations of them.

[1]. Guruprasad, K., B.V. Reddy and M.W. Pandit, Correlation between stability of a protein and its dipeptide composition: a novel approach for predicting in vivo stability of a protein from its primary sequence. Protein Eng, 1990. 4(2): p. 155-61.

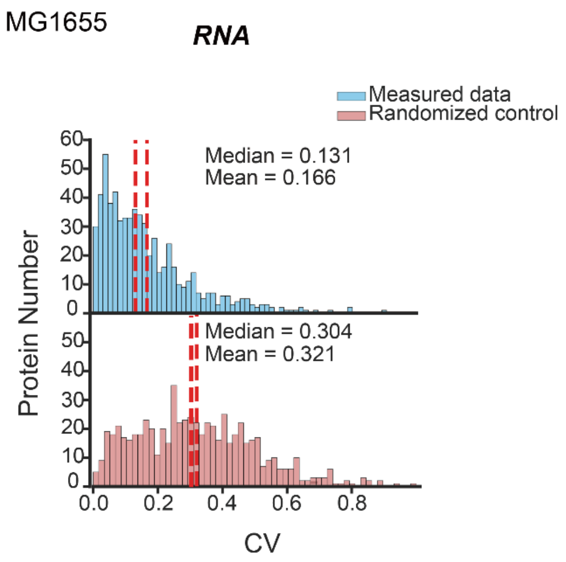

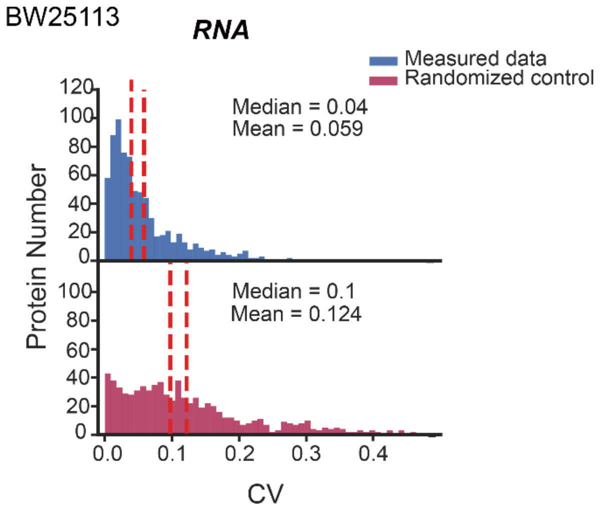

Supplementary Fig. S2: CV within operons of measured data and randomized negative control at RNA level.

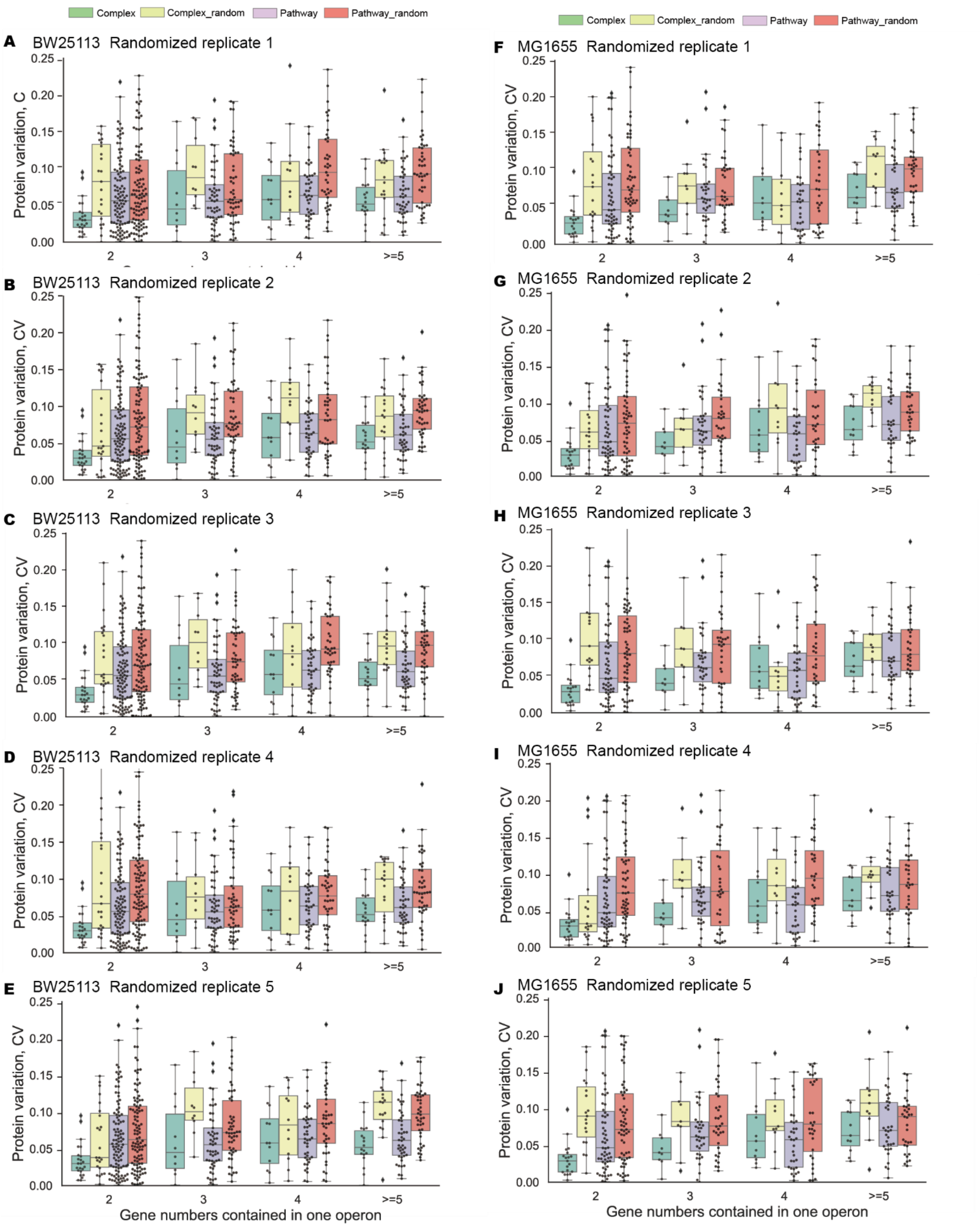

Supplementary Figure S3. Length- and functional-dependence of operon-wise stoichiometry control compare to randomized data. (A-E) Distribution among "Complex", "Complex_random", "Pathway" and

"Pathway_random" groups in BW25113. **(F-J)** Distribution among "Complex", "Complex_random", "Pathway", "Pathway_random" groups in MG1655. In most cases, the CV in "Complex" and "Pathway" groups were lower than corresponding "Complex_random" and "Pathway_random" groups in both strains, indicating real robust signals but not statistical artefacts in operons present in *E. coli.*

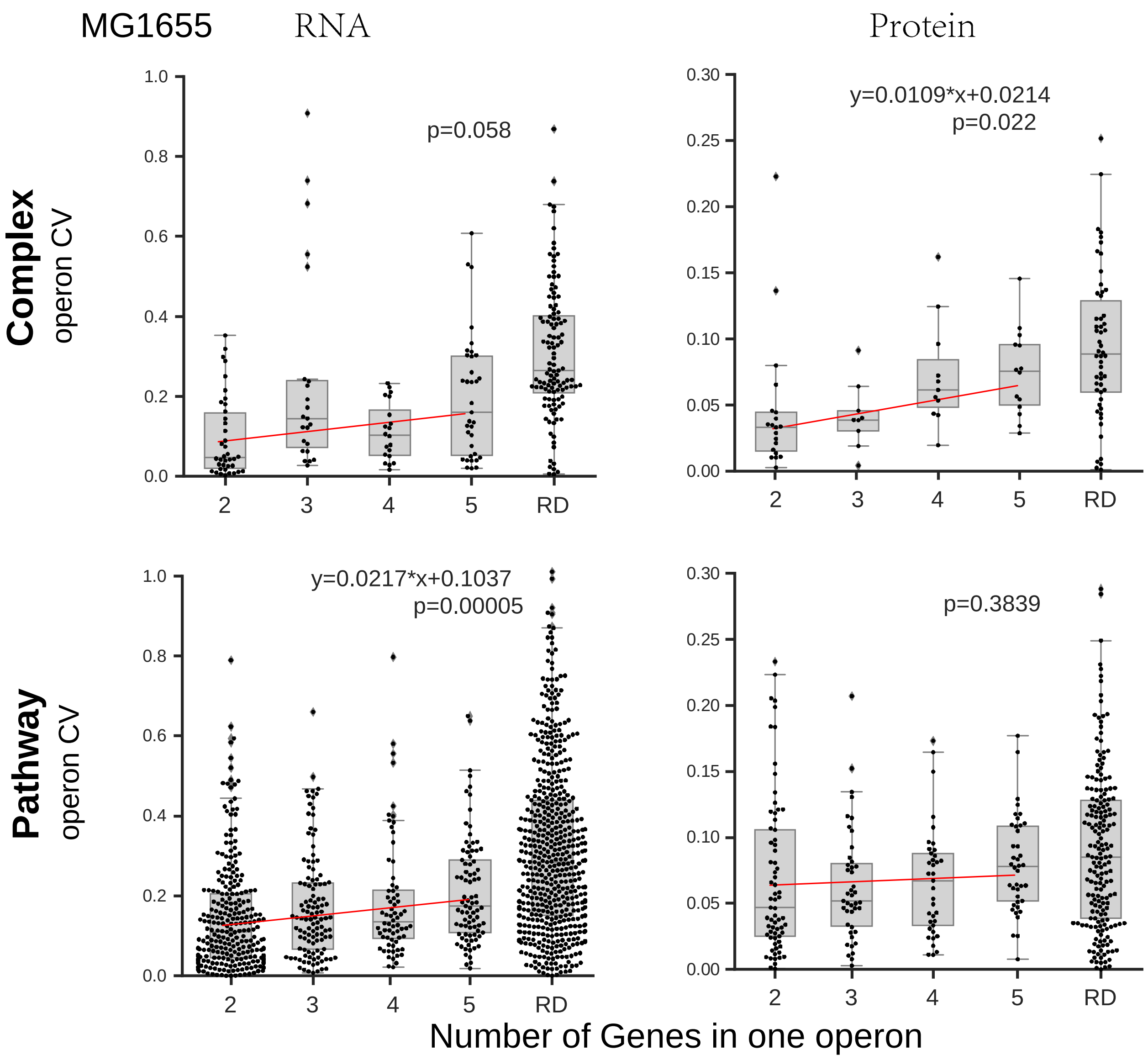

Supplementary Figure S4: Length dependence of the stoichiometry control at RNA and protein levels. Same figure in MG1655 strain, comparable to the Figure 4A.

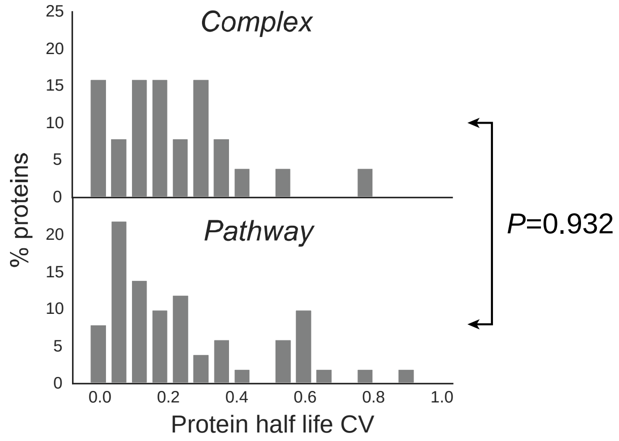

Supplementary Figure S5: Distribution of protein half-life CV in “complex” and “pathway” operons.

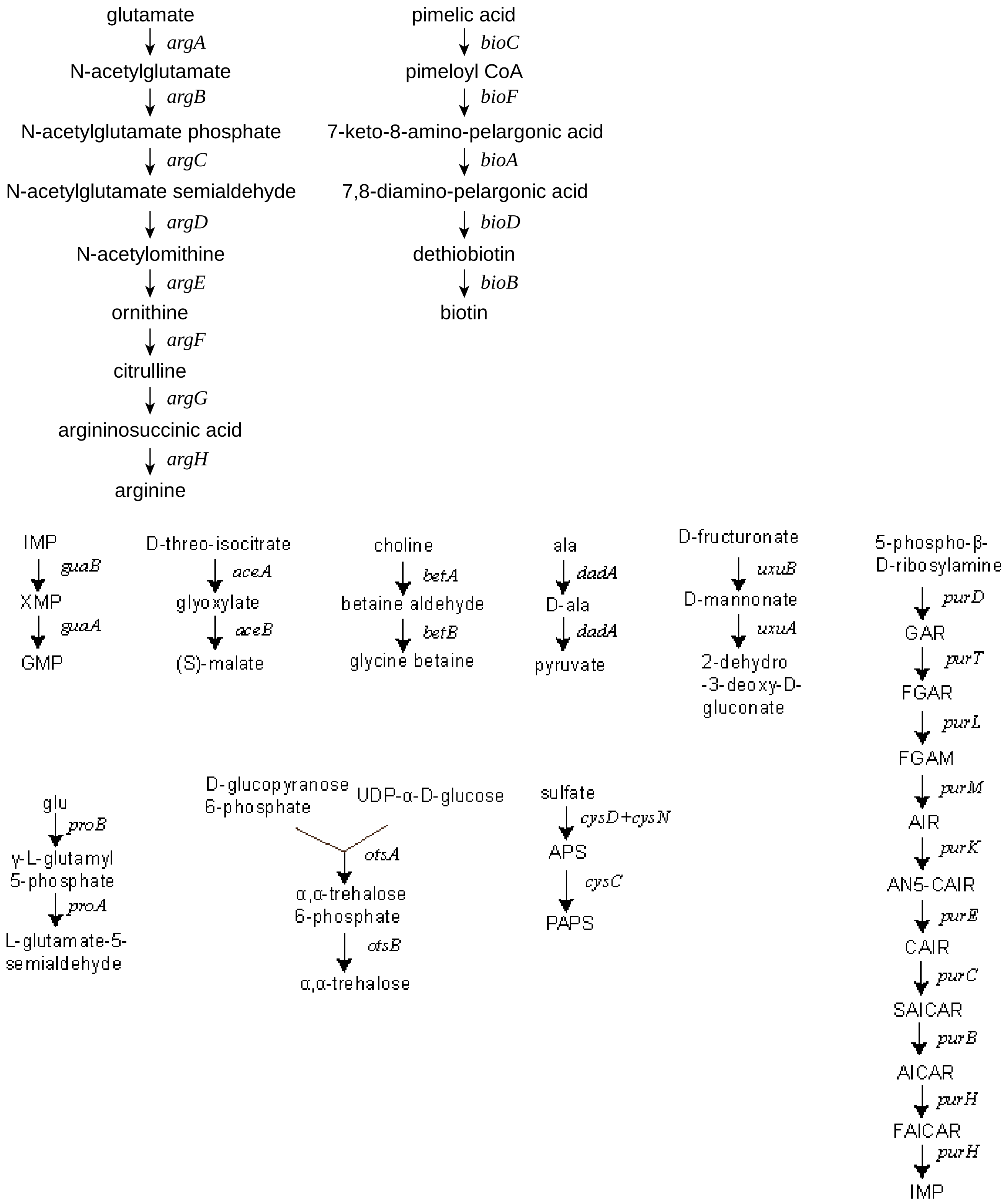

Supplementary Figure S6: Pathways in Figure 5.

Supplementary data| The relationship of submitted raw data

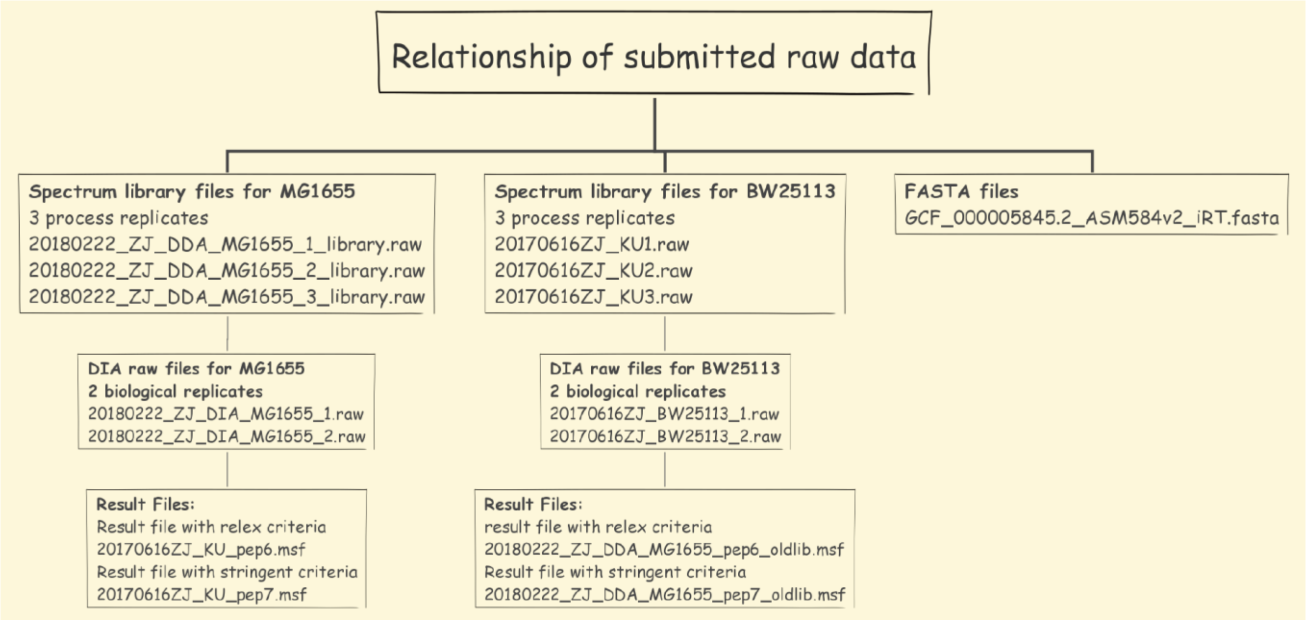

Supplement: FIGURE S1 — Physical and chemical features of quantified and unquantified proteins in E. coli. Panels (A–D) are distributions of protein isoelectric point, instability, protein length, and hydrophobicity. Instability tests a protein for stability, which value above 40 means the protein has a shorter half-life. The Kolmogorov–Smirnov test (KS-test) was used to test the distribution between quantified and unquantified proteins. Significance different were found between quantified and unquantified proteins indicated different physical–chemical characterizations of them. [file Data_Sheet_1.zip › Supplementary Figures.pdf]
