# Supplementary material for: Multifaceted Stoichiometry Control of Bacterial Operons Revealed by Deep Proteome Quantification
Source: Front Genet. 2019 May 24;10:473. doi: 10.3389/fgene.2019.00473 (PMC6544118; doi:10.3389/fgene.2019.00473)
Supplement: FIGURE S1 — Physical and chemical features of quantified and unquantified proteins in E. coli. Panels (A–D) are distributions of protein isoelectric point, instability, protein length, and hydrophobicity. Instability tests a protein for stability, which value above 40 means the protein has a shorter half-life. The Kolmogorov–Smirnov test (KS-test) was used to test the distribution between quantified and unquantified proteins. Significance different were found between quantified and unquantified proteins indicated different physical–chemical characterizations of them. [file Data_Sheet_1.zip › Supplementary. The relationship of submitted raw data.pdf]

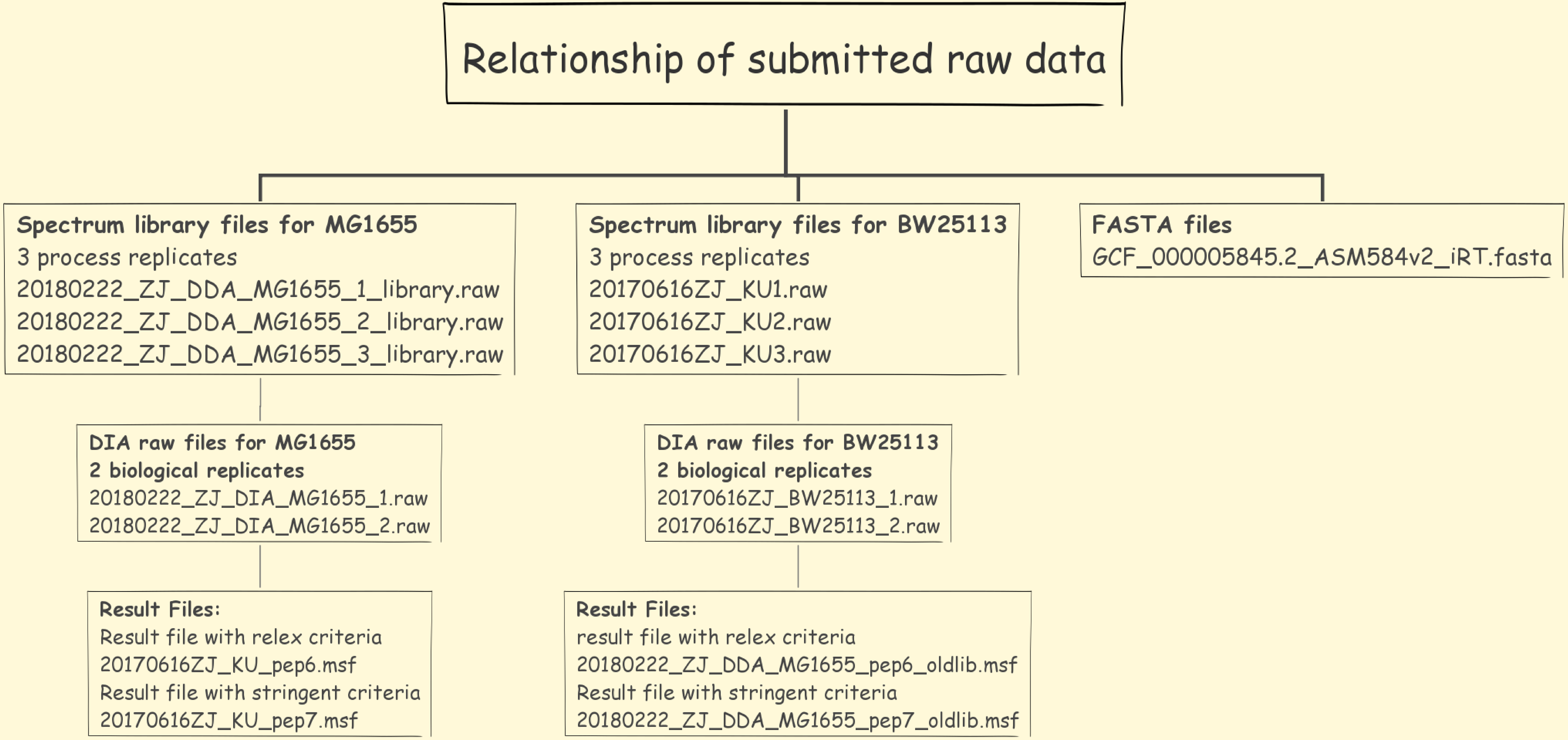
Relationship of submitted raw data
Spectrum library files for MG1655
3 process replicates
20180222_ZJ_DDA_MG1655_1_library.raw
20180222_ZJ_DDA_MG1655_2_library.raw
20180222_ZJ_DDA_MG1655_3_library.raw
Spectrum library files for BW25113
3 process replicates
20170616ZJ_KU1.raw
20170616ZJ_KU2.raw
20170616ZJ_KU3.raw
FASTA files
GCF_000005845.2_ASM584v2_iRT.fasta
DIA raw files for MG1655
2 biological replicates
20180222_ZJ_DIA_MG1655_1.raw
20180222_ZJ_DIA_MG1655_2.raw
DIA raw files for BW25113
2 biological replicates
20170616ZJ_BW25113_1.raw
20170616ZJ_BW25113_2.raw
Result Files:
Result file with relex criteria
20170616ZJ_KU_pep6.msf
Result file with stringent criteria
20170616ZJ_KU_pep7.msf
Result Files:
result file with relex criteria
20180222_ZJ_DDA_MG1655_pep6_oldlib.msf
Result file with stringent criteria
20180222_ZJ_DDA_MG1655_pep7_oldlib.msf
